# Harmonics-assisted optical phase amplifier

**DOI:** 10.1038/s41377-022-01003-3

**Published:** 2022-10-27

**Authors:** Wu-Zhen Li, Chen Yang, Zhi-Yuan Zhou, Yan Li, Yin-Hai Li, Su-Jian Niu, Zheng Ge, Li Chen, Guang-Can Guo, Bao-Sen Shi

**Affiliations:** 1 CAS Key Laboratory of Quantum Information, University of Science and Technology of China, Hefei, Anhui 230026 China; 2 CAS Center for Excellence in Quantum Information and Quantum Physics, University of Science and Technology of China, Hefei, 230026 China; 3 Hefei National Laboratory, University of Science and Technology of China, Hefei, 230088 China

**Keywords:** Nonlinear optics, High-harmonic generation

## Abstract

The change in the relative phase between two light fields serves as a basic principle for the measurement of the physical quantity that guides this change. It would therefore be highly advantageous if the relative phase could be amplified to enhance the measurement resolution. One well-known method for phase amplification involves the use of the multi-photon number and path-entangled state known as the NOON state; however, a high-number NOON state is very difficult to prepare and is highly sensitive to optical losses. Here we propose and experimentally demonstrate in principle a phase amplifier scheme with the assistance of a harmonic generation process. The relative phase difference between two polarization modes in a polarized interferometer is amplified coherently four times with cascaded second-harmonic generation processes. We demonstrate that these amplification processes can be recycled and therefore have the potential to realize much higher numbers of multiple amplification steps. The phase amplification method presented here shows considerable advantages over the method based on NOON states and will be highly promising for use in precision optical measurements.

## Introduction

Phase is one of the most important parameters in both wave optics and quantum mechanics. The relative phase between two light fields in an interferometer or in a superposition state in quantum mechanics is highly significant because the dynamic changes in many physical quantities, including displacement, temperature, and electrical and magnetic fields, can be transduced into changes in the relative phase between light fields or wave functions^1–6^. Therefore, most high-precision measurement tasks can be converted into the measurement of the phase change in a specific physical process, and methods to amplify the phase are highly important for phase measurement resolution enhancement in metrology operations.

One well-known method that is used in quantum optics to realize phase amplification is based on the multi-photon number and path entangled state known as the NOON state ($\left| {N::0} \right\rangle = \left( {\left| {N0} \right\rangle _{AB} + \left| {0N} \right\rangle _{AB}} \right)/\sqrt 2$), which contains *N* indistinguishable particles in an equal superposition, with all particles being in either of the paths *A* or *B*^1^. For interference with NOON states, the phase oscillation is *N* times faster than that for a single photon, and because the effective *N*-photon de Broglie wavelength is $\lambda /N$, this will result in phase super-resolution for high-precision measurements^7^.

Although NOON states can be used for phase amplification and precision metrology applications, it is very difficult to prepare NOON states with high photon numbers; the highest number of the NOON state to be prepared to date^8^ is around 10. Additionally, the detection probability is very low when *N* is large, and a high-photon-number NOON state is highly sensitive to any optical losses experienced by the photons. Therefore, the determination of another phase amplification method that is much more robust than that using NOON states would be highly significant.

Here we propose and experimentally demonstrate, in principle, a new phase amplification scheme based on assistance from harmonic generation processes. In the proposed scheme, the relative phase between two polarization modes in a polarized interferometer is amplified coherently four times based on the cascaded second-harmonic generation (SHG) processes. Furthermore, we experimentally demonstrate that the phase amplification is not determined by the change in the laser frequency, it can still work even after the second-harmonic frequency is converted back to the fundamental frequency via the difference frequency generation (DFG) process, therefore, higher amplification levels could be achieved in an SHG-DFG recycled scheme.

Our proposed phase amplifier is realized with the assistance of a three-wave mixing process (TWM), e.g., SHG, sum-frequency generation (SFG) or DFG. In TWM, the annihilation of two photons serves to create a new photon from a microscopic viewpoint. The interaction Hamiltonian can be expressed as^9^

1 $$\hat H_{{{{\mathrm{eff}}}}}={{{\mathrm{i}}}}\hbar \kappa \left( {\hat a_1\hat a_2\hat a_3^ + - \hat a_1^ + \hat a_2^ + \hat a_3} \right)$$

where $$\hat a_i$$ and $$\hat a_i^ +$$$$\left( {i = 1,2,3} \right)$$ represent the annihilation and creation operators, respectively, of the three interacting photons; in addition, $$\kappa$$ is a constant that is proportional to the second-order susceptibility $$\chi ^{\left( 2 \right)}$$, the pump power and other experimental parameters of the nonlinear crystal used here. In an SHG process, the annihilation of two photons at a fundamental wavelength creates a photon at the second harmonic wavelength; this simple interaction process will lead to new elements being used to manipulate different aspects of light.

This process is inspired by our previous work, in which we realized SHG of light carrying superposed orbital angular momentum modes^10^; in that work, we demonstrated that the orbital angular momentum carried by light is doubled after the SHG process and also found that the relative phase between the superposed orbital angular momentum modes is also doubled in SHG (see Eq. (3) in Ref. ^10^). Two basic questions then naturally occur: can this phase doubling effect be generalized to optical superposed modes in other degrees of freedom, such as the polarization or the optical path? Can the higher phase amplification be realized by using cascaded TWM processes? Our answer to these questions is “yes” in both cases, and in the text below, we will illustrate the basic idea of the scheme and present a detailed proof-of-principle experimental demonstration.

**Fig. 1:**
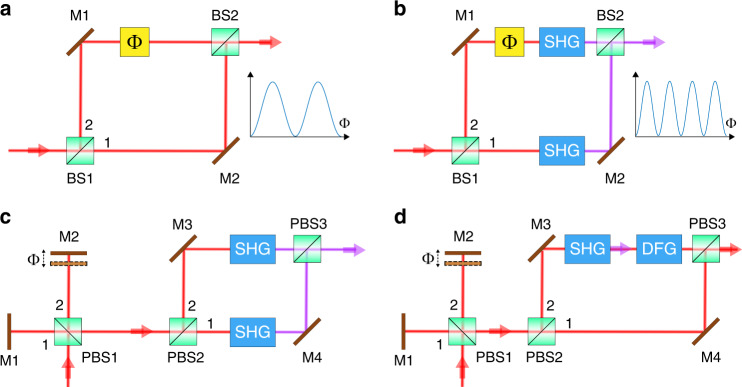
Schematics of the interferometer configurations. **a** MZI. **b** Nonlinear MZI. **c** Nonlinear polarized interferometer. **d** Configuration for phase doubling without frequency doubling

Figure 1 shows a graphical summary of the main concept of this work. Figure 1a shows a schematic of a linear Mach-Zehnder interferometer (MZI) and Fig. 1b shows a nonlinear MZI based on the use of SHG. If we assume that the same phase difference is introduced into both interferometers at the fundamental wavelength, then the speed of variation of the interference fringes in the nonlinear MZI is twice as fast as that in the linear MZI. In wave optics, the interference fields at the output ports of these linear and nonlinear MZIs can be expressed as:

2 $$E_{{{\mathrm{L}}}}\left( \omega \right) = E_1\left( \omega \right) + e^{{{{\mathrm{i}}}}\Phi \left( \omega \right)}E_2\left( \omega \right)$$

3 $$E_{{{{\mathrm{NL}}}}}\left( {2\omega } \right) = E_1\left( {2\omega } \right) + e^{{{{\mathrm{i}}}}2\Phi \left( \omega \right) + {{{\mathrm{i}}}}\Delta \Phi \left( {2\omega } \right)}E_2\left( {2\omega } \right)$$

where $E_1\left( \omega \right)$ and $E_2\left( \omega \right)$ are the two superposed light fields at the beam splitter, and $\Phi \left( \omega \right)$ is the phase difference between these two fields at the fundamental wavelength. $E_1\left( {2\omega } \right)$ and $E_2\left( {2\omega } \right)$ are the two superposed SHG light fields at the beam splitter, and the relationships between the SHG beam and the two fundamental beams are given by $E_1\left( {2\omega } \right) \propto E_1\left( \omega \right)^2$ and $E_2\left( {2\omega } \right) \propto E_2\left( \omega \right)^2$, respectively; and $\Delta \Phi \left( {2\omega } \right)$ is the phase difference between the SHG beams. If the nonlinear interferometer contains *n*^th^ harmonic generation processes, then the output fields from the nonlinear MZI can be expressed as:

4 $$E_{{{{\mathrm{NL}}}}}\left( {n\omega } \right) = E_1\left( {n\omega } \right) + e^{{{{\mathrm{i}}}}n\Phi \left( \omega \right) + {{{\mathrm{i}}}}\Delta \Phi \left( {n\omega } \right)}E_2\left( {n\omega } \right)$$

where $E_1\left( {n\omega } \right)$ and $E_2\left( {n\omega } \right)$ are the two superposed *n*^th^ harmonic light fields at the beam splitter, and the relationships between these *n*^th^ harmonic beams and the fundamental beam are given by $E_1\left( {n\omega } \right) \propto E_1\left( \omega \right)^n$ and $E_2\left( {n\omega } \right) \propto E_2\left( \omega \right)^n$, respectively. $\Delta \Phi \left( {n\omega } \right)$ represents the total phase difference that contains the phase difference of the harmonic beams ranging from the 2nd to the *n*th. These phase differences are caused by different dispersion of the two polarized modes transmitting through different optical elements such as nonlinear crystal, wave plates, polarizing beam splitters (PBSs), etc., and can be kept constant during the experiments. By assuming $E_1\left( \omega \right) = E_2\left( \omega \right)$, Eqs. (3) and (4) clearly indicate that the phase difference $\Phi \left( \omega \right)$ introduced by the fundamental beam is amplified to $n\Phi \left( \omega \right)$ and the light intensity is given by $I_1\left( {n\omega } \right)\left\{ {1 + \cos \left[ {n\Phi \left( \omega \right) + \Delta \Phi \left( {n\omega } \right)} \right]} \right\}$. The dependence on $n\Phi \left( \omega \right)$ rather than $\Phi \left( \omega \right)$ is phase super-resolution: one cycle of $I_{{{{\mathrm{NL}}}}}\left( {n\omega } \right)$ implies a smaller change of the phase difference than one cycle of $I_{{{\mathrm{L}}}}\left( \omega \right)$^1^.

In Fig. 1c, a polarized Michelson interferometer is used instead of the MZI and works in a manner similar to the system in Fig. 1b, but it is much easier to realize experimentally. For the scheme in Fig. 1c, $\Phi \left( \omega \right)$ in Eq. (3) can be adjusted by controlling arm difference in the Michelson interferometer with a modulator or a piezoelectric transducer, while $\Delta \Phi \left( {2\omega } \right)$ is a constant that contains all the phase difference generated in the MZI.

To demonstrate that the phase amplification is not frequency-dependent, we propose and experimentally demonstrate the scheme shown in Fig. 1d. In this scheme, a SHG-DFG module that can double the phase but not change the frequency is inserted in one arm of the MZI, which converts the frequency from $\omega$ to $2\omega$ in the SHG process and from $2\omega$ back to $\omega$ in the DFG process. The interference field at the output ports can be expressed as

5 $$E_{{{{\mathrm{NL}}}}}\left( \omega \right) = E_1\left( \omega \right) + e^{{{{\mathrm{i}}}}2\Phi \left( \omega \right) + {{{\mathrm{i}}}}\Delta \Phi \left( {2\omega } \right)}E_2\left( \omega \right)$$

The phase difference is amplified by 2 times without changing the frequency, which demonstrates that the phase amplification is frequency-independent. Moreover, if the nonlinear conversion efficiencies for both SHG and DFG are high enough, then the output light can enter another SHG-DFG module to realize 4 times phase amplification. Considering that the nonlinear conversion efficiency is dependent on the laser intensity, this scheme could be cascaded more times to achieve much higher amplification levels for the phase difference if a high-intensity laser is used. Some additional recycling methods that can be used to achieve high amplification levels are described in the Supplementary Information.

## Results

To realize interference of the SHG beams, a phase doubling module can be constructed using either a Sagnac loop with a nonlinear crystal^11^ or orthogonally placed nonlinear crystals^12^. These two methods represent better options than the MZI with respect to the phase difference stability because the two polarization components transmit through the same path. In our experiment, the Sagnac loops and orthogonally placed crystals are cascaded to realize interference of two fourth-harmonic (FH) beams and thus demonstrate that the measurement resolution of the phase difference is amplified by four times.

**Fig. 2:**
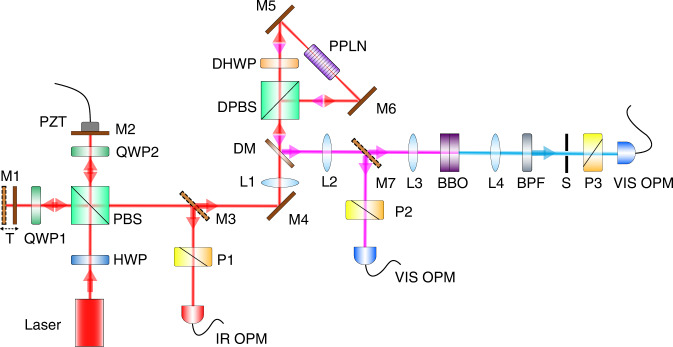
Experimental setup schematic. HWP half-wave plate, QWP quarter-wave plate, DHWP 1560 nm/780 nm dichroic HWP, PBS polarizing beam splitter, DPBS dichroic PBS, M silver-coated mirror, T translation stage, PZT piezoelectric transducer, DM dichroic mirror, BPF 390–10 nm band-pass filter, PPLN periodically poled lithium niobate crystal, BBO β-barium borate crystal, S small hole, P polarizer, IR OPM infrared optical power meter, VIS OPM visible optical power meter, L lens, lenses L1 and L3 are used for focusing and L2 and L4 are used for collimation, and the focal lengths of L1–L4 are 200 mm, 200 mm, 50 mm, and 100 mm, respectively

**Fig. 3:**
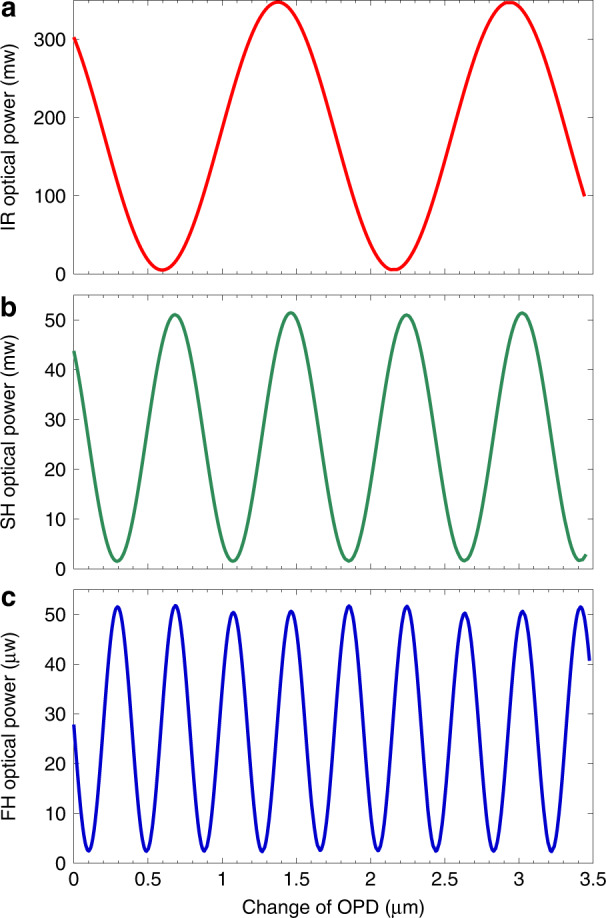
Dependence of optical power after interference on the change in OPD. **a** Fundamental light; **b** SH light; **c** FH light

We used a femtosecond pulsed laser with a centre wavelength of 1560 nm as the fundamental frequency light source to pump the two cascaded SHG modules, as shown in Fig. 2. Full details of the experimental setup are summarized in the Methods section. The interference results for the fundamental, SH and FH beams are shown in Fig. 3a–c, respectively. The *y*-axis in Fig. 3 represents the measured optical power and the *x*-axis represents the change in the optical path difference (OPD). As shown in Fig. 3, the periods of the interference curves of the SH and FH beams are reduced to 1/2 and 1/4, respectively, compared to the fundamental interference curves, which indicates that the phase difference 2π corresponding to one period of the interference curve is amplified to 4π and 8π respectively. Therefore, these results clearly demonstrate that the phase difference for the fundamental beam has been doubled and quadrupled by SHG and FHG, respectively.

Significantly, the three OPDs shown in Fig. 3a–c are all obtained from the same Michelson interferometer, in which the centre wavelength of the light is always 1560 nm. Although the observed phase doubling is accompanied by frequency doubling, the latter is not the reason for the occurrence of the former.

**Fig. 4:**
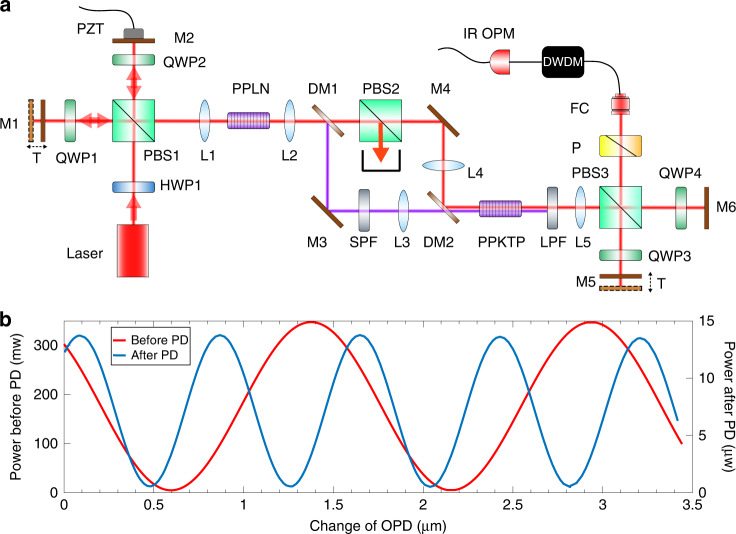
Experimental setup and results. **a** Experimental setup used for phase doubling (PD) without frequency doubling. SPF: 1000 nm short pass filter; LPF: 1400 nm long pass filter; FC: fibre collimator; CH22: channel 22 of a dense wavelength division multiplexing system with a centre wavelength of 1559.79 nm. **b** Measured optical power characteristics of interference before and after phase doubling

To demonstrate that the observed phase doubling is independent of the operating wavelength, we performed a DFG process to convert light with the wavelength of 780 nm back to 1560 nm and thus verify that the phase doubling still exists. Because the output light after both SHG and DFG has the same wavelength as the original input light, phase doubling can be realized by implementing both SHG and DFG in a single arm of the interferometer. In the experiment, the DFG procedure is the inverse process of type-II (yzy) SHG; the setup used is shown in Fig. 4a. We assume here that the states of the horizontally and vertically polarized light after the Michelson interferometer are $\left| {\phi _1,\omega {{{\mathrm{,H}}}}} \right\rangle$ and $\left| {\phi _2,\omega {{{\mathrm{,V}}}}} \right\rangle$, respectively, where $\phi _1$ and $\phi _2$ represent the initial phases, which are dependent on the lengths of the two interferometer arms. After SHG, the state $\left| {2\phi _2,2\omega {{{\mathrm{,V}}}}} \right\rangle$ is generated from $\left| {\phi _2,\omega {{{\mathrm{,V}}}}} \right\rangle$. A dichroic mirror (DM) is used to separate the light at the different wavelengths (1560 nm and 780 nm) into different paths. In the path for the 1560 nm light, a polarizing beam splitter is used to filter out the state $\left| {\phi _2,\omega {{{\mathrm{,V}}}}} \right\rangle$. The two remaining states, $\left| {2\phi _2,2\omega {{{\mathrm{,V}}}}} \right\rangle$ and $\left| {\phi _1,\omega {{{\mathrm{,H}}}}} \right\rangle$, are then combined to perform the DFG operation and the state $\left| {2\phi _2 - \phi _1,\omega {{{\mathrm{,V}}}}} \right\rangle$ is generated. Finally, a polarized Michelson interferometer is used to compensate for the OPD caused by polarization mode dispersion, and a polarizer is used to produce polarized light interference between the states $\left| {2\phi _2 - \phi _1,\omega {{{\mathrm{,V}}}}} \right\rangle$ and $\left| {\phi _1,\omega {{{\mathrm{,H}}}}} \right\rangle$. The interference result is dependent on the phase difference$2\left( {\phi _2 - \phi _1} \right)$, which is doubled compared with the original phase difference $\phi _2 - \phi _1$. The data shown in Fig. 4b confirm that the phase difference is doubled.

## Discussion

In summary, we have presented an attractive method to enable the realization of an optical phase amplifier with the assistance of a TWM process in an interferometer. We achieve a phase difference amplification of four times in the optical phase amplifier by demonstrating the reduction of the period of the interference curve to 1/4 of the original period. This indicates that anything causing the interference curve to change for one period can cause the interference curve to change for 4 periods by using the optical phase amplifier, therefore the resolution of the interferometer is improved by 4 times. We believe the optical phase amplifier presented in this work could be used in numerous precision measurement scenarios, such as the measurements of the optical properties (for example, dispersion and absorption coefficients) of transparent materials^13,14^, other physical quantities such as displacements, angles, and electrical and magnetic fields, which can be transduced into changes in an optical path length^15^, or interference-based imaging for enhanced image resolution^16^, etc. In principle, the proposed optical phase amplifier that is based on SHG in our experiment could be generalized to high-order harmonic generation processes^17,18^.

Although a maximum phase amplification of four is achieved in the experiments presented here, a SHG-DFG recycling scheme described in detail in the supplementary material that provides the potential to achieve higher phase amplification levels is proposed too. In our experiment, the FHG efficiency of 0.07% and the DFG efficiency of ~2% are low because of the low pump power and mismatched bandwidths (the bandwidths of two input lights and the acceptance bandwidth of the crystal should be matched to obtain high DFG efficiency). Fortunately, under suitable experimental conditions (with a high-intensity pump laser and a proper crystal), the power conversion efficiency of both SHG and DFG can be improved up to 80%^19,20^, therefore, much higher phase amplification levels could be achieved in principle. If the efficiencies of the nonlinear processes are high enough, the amplification level is expected to surpass the highest amplification levels achieved using NOON states. Moreover, the method presented in this work is much more robust than that based on NOON states because the latter needs to take a long time in the coincidence measurement by using single-photon detectors. While in this work, the high-speed photodiode can be used in the real-time measurement, the interference curves are close to perfect sine curves, error bars and fitting are not necessary which are important for processing interference results of NOON states^1,21,22^.

We realize that there are various studies in phase estimation^23–26^. We would like to clarify the obvious differences between the present work and those schemes. Previous literatures focus on the phase estimation of non-classical light sources in a nonlinear interferometer. The light source generated is far below the threshold of nonlinear processes and the mean photon number is very small, so these works study the phase estimation in the quantum regime and one can achieve Heisenberg limits (1/*N*) for phase estimation. Besides, the phase estimation is based on the coincidence measurement by using single-photon detection or homodyne detection. While in our present scheme, all light sources are lasers, which are above the threshold of the nonlinear processes, the mean photon number is very large, a high-speed photodiode can be used for measuring the interference curve, and the limits for phase measurement are the standard quantum limit ($1/\sqrt N$). The distinct feature of our present scheme is the phase super-resolution: after our phase amplifier, a change in light intensity in one cycle corresponds to a much smaller change in phase difference than that before the phase amplification. The work presented here will revolutionize our understanding of nonlinear interference and may open new avenues for interference-based precision metrology.

## Materials and methods

### FHG experiment

A schematic of the experimental setup is shown in Fig. 2. The input light is a pulsed laser beam with a centre wavelength of 1560 nm, a repetition frequency of 80 MHz and a pulse width of 150 fs. The linearly polarized beam is transformed into a 45°-polarized beam using a half-wave plate (HWP), and thus the input beam is divided equally into two orthogonal linearly polarized light beams by the PBS. In each arm of the Michelson interferometer, a group composed of a mirror and a quarter-wave plate (QWP) can transform the beam’s polarization state into its orthogonal state^10^, which causes the light to exit the interferometer from the other port of the PBS. Mirror M1 is fixed on a translation stage that is used to perform rough adjustments of the OPD between the two arms. Mirror M2 is fixed on a piezoelectric transducer (PZT) that is used to enable fine adjustment of the OPD within a 3.5 μm range; the PZT is driven using an amplified triangular wave signal.

In the first SHG module, a periodically poled lithium niobate (PPLN) crystal with a length of 5 mm is used. The PPLN crystal satisfying the type-0 (zzz) quasi-phase-matching condition only responds to the vertical polarization of light. Here, a Sagnac loop with a dichroic HWP inserted is used to realize the SHG process for both vertical and horizontal polarizations, which was demonstrated in our previous work^11^.

In our experiment, the average pump power in each arm is 135 mW which is limited by the optimum output power of the femtosecond laser; SH light with a power of 30 mW at a centre wavelength of 780 nm is generated in each polarization state. The second SHG module is composed of a crystal group that comprises two orthogonally glued type-Ι (ooe) birefringence phase matching (BPM)-type β-barium borate (BBO) crystals. Each BBO crystal has a thickness of 0.5 mm and a phase-matching angle of ~30°. The two 780 nm orthogonally polarized light beams are frequency-doubled by the two orthogonally placed BBO crystals. Each polarization state of the 390 nm SH light has an optical power of 95 μW. Therefore, the two orthogonally polarized 1560 nm beams can be frequency-doubled twice sequentially by cascading two SHGs, and the resulting 390 nm light can be considered to be the FHG output from the input 1560 nm laser beam.

The interference of the FH orthogonal polarization states can be observed after the light passes through a 45° polarizer. An optical power meter is used to record the change in the power that occurs when the OPD in the Michelson interferometer is scanned using the PZT driven by the amplified triangular wave. When we observed and recorded the interference of the fundamental frequency light or the SH light, mirror M3 or M7 was inserted, respectively, to separate the light beam from its original optical path; otherwise, these two mirrors are not present in the optical path.

### DFG experiment

In the experiments performed to demonstrate that the phase amplification process is independent of the operating wavelength, we introduced the DFG process, as illustrated in Fig. 4a. In this experiment, we used the same Michelson interferometer configuration and the same PPLN crystal for frequency doubling used in the FHG experiment described earlier. Here, the Sagnac loop is not used, so the crystal only responds to the vertical polarization of light. DM1 transmitted the 1560 nm light and reflected the 780 nm light, while DM2 had the opposite characteristics. The crystal that was used for DFG was a type-II (yzy) periodically poled potassium titanyl phosphate (PPKTP) crystal with a length of 10 mm. A PBS and a short pass filter (SPF) were used to filter out the 1560 nm light, thus suppressing the background noise from the difference frequency light and improving the visibility of the interference. The second Michelson interferometer was used to compensate for the OPD caused by polarization mode dispersion. Because the spectrum of the 1560 nm light after SHG and DFG was changed when compared with the original spectrum, a dense wavelength division multiplexing system was used as a narrow band filter (full width at half maximum of 200 GHz) to improve the interference visibility.

## Supplementary information

Harmonics-assisted optical phase amplifier
