# Supplementary material for: Harmonics-assisted optical phase amplifier
Source: Light Sci Appl. 2022 Oct 27;11:312. doi: 10.1038/s41377-022-01003-3 (PMC9613983; doi:10.1038/s41377-022-01003-3)
Supplement: Supplementary file 1 — Harmonics-assisted optical phase amplifier [file 41377_2022_1003_MOESM1_ESM.docx]

Supplementary Information for

**Harmonics-assisted optical phase amplifier**

Wu-Zhen Li^1, 2, 3, 4^, Chen Yang^1, 2, 3, 4^, Zhi-Yuan Zhou^1, 2, 3, 5^, Yan Li^1, 2, 3^, Yin-Hai Li^1, 2, 3^, Su-Jian Niu^1, 2, 3^, Zheng Ge^1, 2, 3^, Li Chen^1, 2, 3^, Guang-Can Guo^1, 2, 3^ and Bao-Sen Shi^1, 2, 3, 6^

*^1^* CAS Key Laboratory of Quantum Information, University of Science and Technology of China, Hefei, Anhui 230026, China

^2^ CAS Center for Excellence in Quantum Information and Quantum Physics, University of Science and Technology of China, Hefei 230026, China

^3^ Hefei National Laboratory, University of Science and Technology of China, Hefei 230088, China

^4^ These two authors contributed equally to this article.

^5^ [zyzhouphy@ustc.edu.cn](mailto:zyzhouphy@ustc.edu.cn)

^6^ [drshi@ustc.edu.cn](mailto:drshi@ustc.edu.cn)

1. Data acquisition and processing


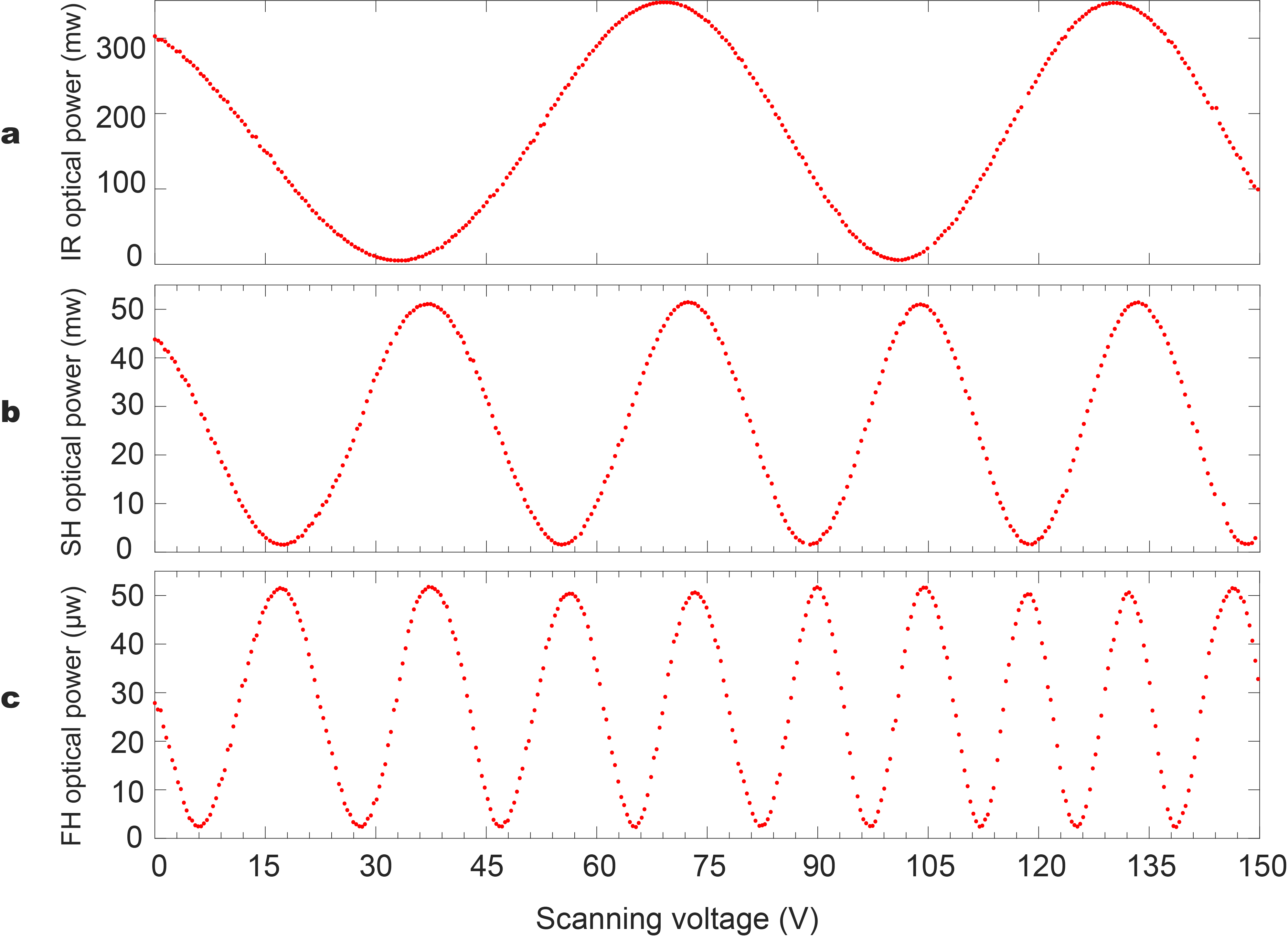


Fig. S1 Raw data with maximum interference visibility.

In the experiment, we studied the phase amplification effect of the second and fourth harmonic generation in a Michelson interferometer. Fig. S1 shows the raw interference data recorded by an optical power meter (OPM) at the three positions of the interferometer. We first use the mirror M3 in Fig. 2 (in the main text) to separate the fundamental frequency beams, and then we record the polarized light interference results using an IR OPM. The measured optical power of the fundamental frequency beams against the PZT scanning voltage is shown in Fig. S1a. In the scanning voltage range of 0 to 150 V, the optical power changes by ~2.25 periods, which indicates that the optical phase difference between the two arms of the interferometer transforms by ~ 4.5π. After recording the results based on fundamental pulse light, we removed M3 to perform the SHG experiment. Then we use M7 to separate the SHG beams, and the optical power after interference is given in Fig. S1b. For the SHG beams, the interference intensity changes ~ 4.5 periods; therefore, phase difference changes by 9π within the scanning voltage range of 150 V. We then remove M7 to perform FHG experiments based on the SHG beams, and the optical power after interference between the two FHG beams is also recorded by the VIS OPM; the raw interference data for FHG beams are shown in Fig. S1c.

Since the relationship between the scanning voltage and the displacement of the PZT is nonlinear, the arm difference ΔL does not change linearly with the voltage of the PZT. Thus, from Fig. S1, we find that the changing period of the optical power of interference with respect to the scanning voltage is a variable. In order to unify the changing period of the optical power, we need to convert the scanning voltages into changes of the optical path difference (OPD), as shown in Fig. 3 in the main text. We first perform function fitting on the experimental data in Fig. S1 to obtain an accurate function relationship between changes of OPD and voltages, and then we make use of the function to convert the scanning voltages into the corresponding changes of OPD.

In our experiment, we collected ~ 320 data points for each interference result within the 150 V scanning range. The curves shown in Fig. 3 in the main text are the results of the direct connection of the data points.

1. Scheme of achieving high amplification times


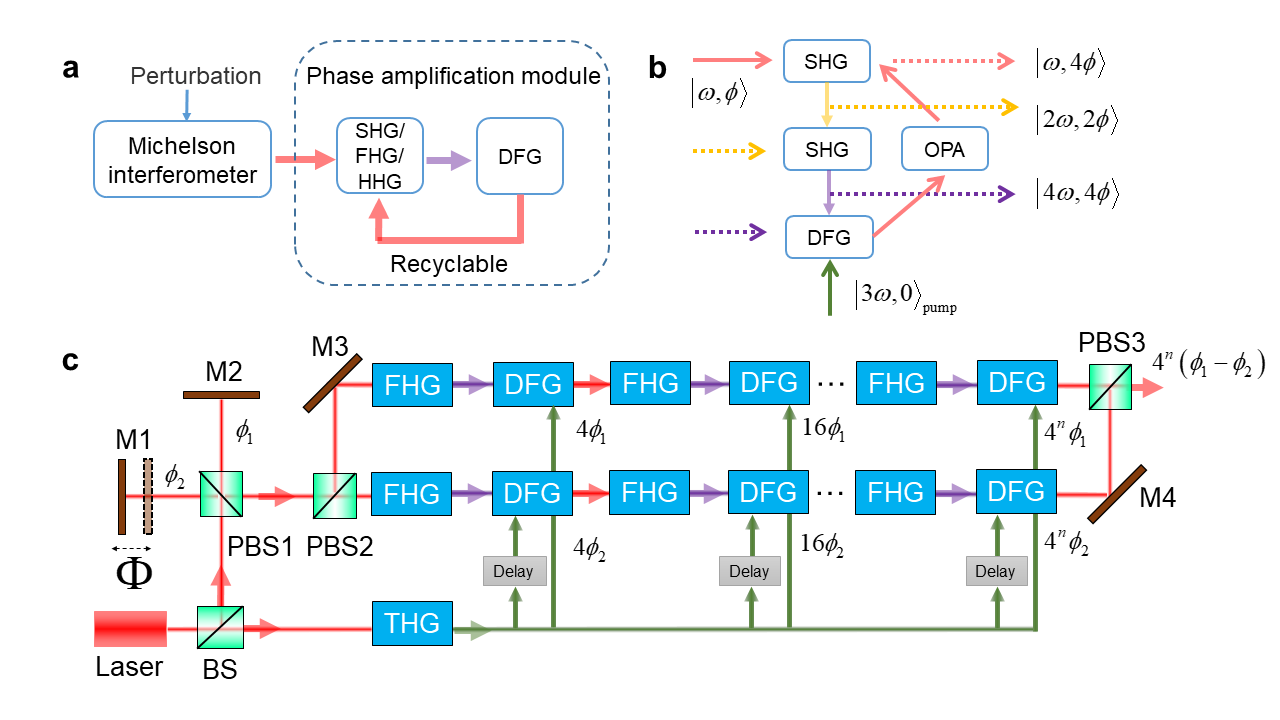


Fig. S2. Schematic of the recyclable phase amplification module. **a** A basic flow chart. **b** A schematic of the FHG-DFG recycling module. **c** An experimental scheme to realize FHG-DFG recycling. The fundamental, FH, and pump lights are marked red, purple, and green, respectively.

In the main text, we present a scheme of a phase amplifier that can amplify the phase difference in an interferometer by 4 times. We also mention that higher amplification times can be obtained by using a recycling scheme introduced in the following.

A basic flow chart is shown in Fig. S2a. Any changes in physical quantities affecting the optical path length can be sensed and transformed into changes in phase difference by a Michelson interferometer. The phase difference can be amplified by the optical phase amplifier that is formed by harmonic generation modules and difference frequency generation (DFG) modules. The amplification is the *n*-th power of the amplification in one cycle, where *n* represents the number of cycles. This flow chart includes the situation presented in the main text, where the SHG module and DFG module are cascaded. To improve the order of harmonic waves, one can cascade more SHG modules except by using high harmonic generation materials. For second-order nonlinear crystals, the complex high-order nonlinear processes are relatively weak; therefore, the noise is easy to filter.

In Fig. S2b, we present a FHG-DFG scheme where two SHG modules are cascaded and used as a fourth-harmonic generation (FHG) module. The advantage of FHG is that the high-efficiency DFG of type-0 quasi-phase-matching can be utilized. In the SHG-DFG scheme that is demonstrated in the main text, the DFG should be based on the type-II phase-matching condition, where the efficiency is much lower than that of type-0 phase-matching. In the recycling module shown in Fig. S2b, the input or output light can not only work at the fundamental frequency $\omega$, but also at SH frequency $2\omega$ or FH frequency $4\omega$, which can be flexibly designed according to the working waveband of the interferometer and the detector. A simple method to realize the recycling is to cascade the combination of FHG and DFG modules, as shown in Fig. S2c.

Unlike the scheme in the main text, an extra pump beam should be introduced in the DFG (and OPA if it is used) modules, and the extra pump beam can introduce a random phase on the DFG light. Therefore, the recycling must be implemented on both two paths and the pump beams should come from the same laser to ensure that the random phase is introduced on both paths and no extra random phase difference is generated. An easy pump scheme is proposed as follows. One can split a part of the fundamental light to implement the third-harmonic generation (THG) process (the THG can be implemented by combining an SHG process and a sum-frequency generation process) and use the THG light with a frequency of $3\omega$ as the pump light of the DFG modules. In a DFG module, the input lights are an FHG signal light ($4\omega$) and a THG pump light ($3\omega$), and the output light has the fundamental frequency $\omega$. Therefore, the output can enter the next FHG-DFG module to realize higher amplification. Significantly, the phase delay between the THG pump light in two paths should be carefully adjusted to make the phase difference generated in the two DFG processes zero.

To increase the number of cycles, the loss in the loop should be low and the nonlinear efficiency should be high. In our experiment, the pulsed pump beam has a full width at half maximum (FWHM) of 150 fs and energy below 5 nJ. Since the frequency conversion efficiency of the ultrashort pulse is limited by low pump energy, bandwidth mismatch (the bandwidths of the input lights and the acceptance bandwidth of the crystal should be matched to obtain high efficiency), and group velocity mismatch in the crystal, the FHG efficiency obtained in our experiment is less than 0.1%. However, high FHG efficiency can be easily achieved by choosing appropriate pump light sources and nonlinear crystals.

For example, in Ref. 1, the FHG energy efficiency of 46.5% was obtained at the fundamental intensity of 1.5 GW cm^-2^, where a large aperture Nd: glass laser that emits high energy pulses is used as the pump light source and an integrated conversion cell consisting mainly of two KDP crystals is used as the nonlinear material. Besides, the synchronously pumped scheme and the optical parameter amplification (OPA) technique can be used to enhance the optical power of the fundamental light generated from the DFG process. For example, in Ref. 2, by synchronizing the signal and pump pulses, the picosecond mid-infrared idler radiation with a high average power (>3.6 W) and high conversion efficiency (maximum pump-to-DFG power conversion 78%) was obtained from the single-pass DFG system. In 2015, Lin Xu et al. reported a high-energy picosecond OPA with an overall conversion efficiency of 45%, where the gain of signal light can reach 79 dB for low seed powers ^3^. Therefore, if a high-power sub-nanometer laser is used as the pump source, which is easily obtained by utilizing chirped pulse amplification (CPA) technology, suitable nonlinear crystals are used for FHG and DFG, and OPA technique is optionally used to amplify the lights after the DFG processes, then, a high-energy pulse can be obtained after one cycle and has sufficient energy to realize FHG-DFG processes in the next cycle.

A phase amplifier based on FHG can achieve 4 times amplification of phase difference. Therefore, the two cycles scheme of FHG-DFG-FHG can realize 16 times amplification, which can easily surpass the highest amplification levels achieved using NOON states (the highest number of the NOON state to be prepared to date is 10, which is reported in Ref. 4). The cycling scheme proposed here has the potential to realize higher phase amplification, which could be verified in future experiments.

**Reference**

1 Bruneau, D., Tournade, A. M. & Fabre, E. Fourth harmonic generation of a large-aperture Nd:glass laser. *Appl. Optics* **24**, 3740-3745 (1985).

2 Murray, R. T., Runcorn, T. H., Kelleher, E. J. R. & Taylor, J. R. Highly efficient mid-infrared difference-frequency generation using synchronously pulsed fiber lasers. *Opt. Lett.* **41**, 2446-2449 (2016).

3 Xu, L., Chan, H. Y., Alam, S. U., Richardson, D. J. & Shepherd, D. P. High-energy, near- and mid-IR picosecond pulses generated by a fiber-MOPA-pumped optical parametric generator and amplifier. *Opt. Express* **23**, 12613-12618 (2015).

4 Wang, X. L. *et al.* Experimental ten-photon entanglement. *Phys. Rev. Lett.* **117** (2016).
